# Not just heard, but judged: a multidimensional perspective on auditory attention in everyday life

**DOI:** 10.3389/fnrgo.2026.1693662

**Published:** 2026-02-23

**Authors:** Silvia Korte, Martin G. Bleichner

**Affiliations:** 1 Translational Psychology, Department of Psychology, University of Oldenburg, Oldenburg, Germany; 2 Research Center for Neurosensory Science, University of Oldenburg, Oldenburg, Germany

**Keywords:** auditory attention, distraction, everyday life, relevance, salience

## Abstract

This review examines how listeners evaluate sounds in everyday contexts and how auditory attention research has approached this process. While experimental paradigms have yielded important insights into auditory processing, their constructs often rely on task-specific definitions that may not fully reflect how sounds are perceived and interpreted outside the laboratory. We argue that auditory evaluation is shaped by the interaction of acoustic properties, affective tone, task demands, and contextual framing. To account for this, we propose a multidimensional framework based on arousal, valence, and context, which enables a more flexible characterization of how sounds are judged in everyday listening. We also examine how different methodological approaches highlight distinct facets of this evaluative process. By focusing on the conditions under which sounds are experienced, this review contributes to a more integrative understanding of auditory attention across both controlled and naturalistic settings.

## 1 Introduction

Sound is a continuous part of the human environment and can exert multiple effects on a person over the course of a day, influencing mood, concentration, and general wellbeing. Because acoustic events interact with a person's ongoing bodily and affective state, their impact varies from moment to moment, sometimes subtly and sometimes quite noticeably.

Understanding these effects is complicated by the fact that different research traditions approach the influence of sound on the individual from different levels of description. Physiological studies examine bodily activation, auditory neuroscience describes large-scale neural dynamics, environmental acoustics focuses on physical sound properties, cognitive psychology considers task performance, and social or environmental psychology investigates subjective evaluations. Each of these perspectives offers valuable insights, yet they rely on distinct terminology, conceptual assumptions, and observational scales. When these levels are blended without care, acoustic properties can be treated as if they directly cause psychological states, neural measures can be mistaken for cognitive constructs, and subjective reports can be interpreted as physiological indices.

Our aim in this article is to make the relations between these traditions transparent: to show where insights can be meaningfully connected, where translations between levels of description are limited, and why maintaining conceptual boundaries is crucial for understanding everyday listening. Concretely, we use the everyday distinction between “relevant” and “distracting” sounds as a guiding case and stress that these labels are not fixed stimulus properties but context-dependent evaluations that emerge from interactions between acoustic structure, attentional selectivity, and listener state. Accordingly, this review does not propose a new mechanistic model of auditory perception or attentional selection; instead, it provides a conceptual mapping that clarifies how perceptual, attentional, and evaluative levels relate, and what each can (and cannot) explain in real-world listening. We first distinguish stimulus-driven factors (e.g., salience), distraction mechanisms, and contextual determinants of relevance, and then synthesize them into an integrative perspective with methodological implications for triangulating acoustic, behavioral, subjective, and neural measures.

One example that illustrates the necessity for more clarity is auditory distraction. Distraction is often treated as a single phenomenon, yet it is described differently across fields: as a behavioral cost, a physiological load, a neural perturbation, an environmental mismatch, or an aversive experience. Its real-world consequences are significant, auditory distraction contributes to errors in air-traffic control (Giraudet et al., 2015), increases stress in hospital staff (Morrison et al., 2003), impairs learning in classrooms (Massonnié et al., 2022), and diminishes well-being in individuals with heightened sound sensitivity, including those with autism (Stiegler and Davis, 2010). Examining distraction through the lens of distinct descriptive levels shows how easily interpretations can be conflated — and why a clearer conceptual structure is needed when studying everyday sound perception.

### 1.1 Key concepts

To examine how people perceive and evaluate everyday sounds, it is necessary to clarify the terms that structure this inquiry. In both experimental and theoretical literature, the concepts of attention, distraction, and relevance are used to describe different facets of auditory processing. These terms are often defined operationally, based on task performance, rather than in relation to the listener's interpretive experience.

Auditory attention refers to the process by which listeners selectively prioritize certain acoustic information over others, depending on current goals, expectations, and contextual demands. It includes both voluntary (top-down) and involuntary (bottom-up) mechanisms and is widely recognized as a dynamic, fluctuating process that responds to internal and external cues (e.g., Shinn-Cunningham and Best, 2008; Parmentier et al., 2010).

Attentional shifts refer to changes in the focus of attention from one stimulus or location to another. These shifts can be voluntary (top-down), such as when a listener deliberately tunes in to a conversation, or involuntary (bottom-up), as when attention is captured for example by the sudden occurance of a sound. In both cases, attentional shifts reflect the dynamic reallocation of cognitive resources in response to changing internal goals or external stimuli (e.g., Corbetta and Shulman, 2002; Knudsen, 2007).

Distraction occurs when attention is drawn away from an ongoing task or goal. In experimental paradigms in psychology, this is typically measured as a performance cost following the presentation of a task-irrelevant stimulus (Hughes, 2014). Such an operationalization captures one observable consequence of distraction, but it does not account for instances where attention is diverted without any measurable change in task performance (Berti et al., 2004). Importantly, in naturalistic settings, distraction also includes the subjective experience of being interrupted, overwhelmed, or otherwise pulled away from intended activity (e.g., Massonnié et al., 2022). Thus, distraction is not only a behavioral outcome but an evaluative judgment that a particular sound has interfered with attentional focus (Hughes, 2014).

Relevance refers to the degree to which a sound is judged as meaningful, useful, or important in a given context. Relevance is not an inherent property of a sound but a situational and subjective assessment shaped by the listener's goals, affective state, and social or environmental context (Asutay and Västfjäll, 2012; Siegel and Stefanucci, 2011; Debnath and Wetzel, 2022). Importantly, the same sound may be judged as irrelevant in one moment and relevant in another.

Salience refers to the perceptual prominence of a sound (i.e., how strongly it stands out from its background based on acoustic or statistical properties such as intensity, novelty, or spectral change). Salience is a stimulus-derived feature that increases the likelihood of an attentional shift. Importantly, salience describes the noticeability of a sound and not its meaning: a salient event may capture attention without being evaluated as relevant, and a less salient event may also capture attention and be interpreted as important depending on the listener's goals or expectations (e.g., Kaya and Elhilali, 2014; Symons et al., 2024).

In this review, we focus on the concepts of distraction and relevance because they represent the most commonly assigned roles in experimental research on auditory attention—typically as oppositional categories, where one sound is designated as a distractor and another as a target (e.g., Berti et al., 2004; Schröger et al., 2000; Escera et al., 2000; Horváth et al., 2008; Näätänen et al., 1993). By re-examining these categories in relation to real-world listening, we explore where they remain useful and where they may fall short in capturing real-world experience.

## 2 A multidimensional view of auditory attention

Auditory attention in everyday life emerges from the interplay of multiple factors, including the physical properties of sound, its potential to disrupt ongoing activity, the surrounding context, and the listener's internal state. In this section, we outline a multidimensional view of auditory experience by examining four interconnected aspects: salience, distraction, context, and temporal dynamics. Together, these dimensions provide the conceptual foundation for the methodological considerations discussed in Section 3.

### 2.1 What makes a sound salient?

One way to understand how sounds are perceived, is to consider the salience of the sound: the extent to which acoustic properties make them more or less likely to capture attention. The acoustic features of a sound play a pivotal role in determining its perceptual salience (e.g., Kaya and Elhilali, 2014; Symons et al., 2024; Bouvier et al., 2023). Understanding what makes a sound stand out, and what happens in the brain when it does, is central to explaining how a sound is evaluated.

The physical characteristics of sound strongly influence in how far they can attract attention. Although intensity is frequently cited (e.g., Schadow et al., 2007; Bach et al., 2008), research increasingly highlights a combination of psychoacoustic features such as sharpness, roughness, fluctuation strength, and tonality as more reliable predictors of attentional capture and how the sound is consequently evaluated (Sottek and Genuit, 2005). For example, Rimskaya-Korsakova et al. (2022) showed that even sounds with similar intensity can differ markedly in their salience, depending on these finer-grained properties.

Computational models further support the role of sound charactristics in determining salience. The auditory salience map proposed by Kaya and Elhilali (2014), based on spectrotemporal contrast, intensity and frequency, predicts behavioral responses and eye movements. In a similar vein, Huang and Elhilali (2020) demonstrated that salient background events could disrupt neural phase-locking to a foreground stream, indicating that acoustic features alone can drive attentional capture. Massoudi et al. (2013) recorded from the auditory cortex in monkeys and found that core features like frequency and intensity were robustly encoded in a largely task-invariant fashion. Even in the absence of explicit task relevance, these acoustic signatures were preserved at the neural level, showing a bottom-up encoding of auditory salience.

These findings are in line with Sokolov's *orienting reflex model* (Sokolov, 1963), which describes how the brain maintains a representation of environmental regularities and generates an alerting response when a stimulus violates this internal model. The orienting reflex includes physiological changes such as heart rate deceleration and pupil dilation, as well as a temporary reallocation of attention. Importantly, this reflex provides a neutral mechanism for detecting change and preparing the system for further evaluation.

Predictive coding models help formalize this process by suggesting that the brain continuously generates predictions about incoming auditory input and updates its internal model when these predictions are violated (Hughes and Marsh, 2020; Winkler, 2007). Salient events are characterized by high prediction error, leading to heightened sensory processing and, potentially, attentional shifts. Within this framework, salience is not solely a property of the sound but a relational outcome between the sound and the listener's expectations.

Taken together salience is an important factor for understanding sound processing. A sudden noise might capture and redirect attention. However, salience alone is not sufficient to understand how sounds are processed and evaluated. The next sections examine the downstream consequences of salient events.

### 2.2 What makes a sound distracting and to whom?

Although salient sounds may draw attention, salience alone does not make a sound distracting. Many events are noticed without consequence, whereas others become disruptive because they interfere with ongoing goals. Distraction arises when a sound, independent of its salience, causes an unvoluntary attentional shift that conflicts with the current activity. This section explores how such goal-conflicting attentional shifts are shaped by task demands, individual listener characteristics, and perceptual mechanisms. It draws on multiple theoretical perspectives to illustrate that distraction is the outcome of a dynamic and context-sensitive attentional system.

Current theories differ in how they explain the chain of events from noticing a sound to being disrupted by it. What turns a perceptually noticeable event into a behavioral or experiential interference depends on how it interacts with cognitive goals and attentional engagement. Two influential perspectives illustrate different ways of conceptualizing this process. The duplex-mechanism account (Hughes, 2014) highlights broad outcome categories, whereas the three-phase model by Escera et al. (2000) specifies the temporal sequence of underlying cognitive operations.

The *duplex-mechanism account* (Hughes, 2014) proposes that some sounds trigger attentional capture without measurable behavioral cost, while others result in clear performance decrements. This account highlights an important distinction between changes in attentional focus and observable task performance: a sound can draw on attentional resources (e.g., by briefly orienting attention away from the task) without this necessarily translating into slower responses or more errors.

The *three-phase model of involuntary attention and distractibility* (Escera et al., 2000) offers a more detailed sequence of how distraction unfolds. According to this model, auditory deviance elicits a cascade of cognitive processes. In this model, deviance is detected pre-attentively, leading to an involuntary orienting of attention away from the primary taks and toward the deviant sound. This is followed by a voluntary return to the primary task. This structure emphasizes that distraction is not a failure of control but a dynamic reallocation of resources in response to potentially significant change: The task is interrupted to evaluate the relevance of the sounds, if deemed irrelevant or inconsequential, the attention is redirected to the primary task.

Whether a sound disrupts performance depends not only on its acoustic properties, but also on what the listener is doing at the time. Models of task load and resource allocation help explain when ongoing activities make us more—or less—susceptible to distraction. One of the most robust predictors of distraction is the complexity or nature of the task at hand. According to the *central bottleneck model*, task-relevant processing occupies a limited-capacity system (Fagot and Pashler, 1992), and under high cognitive load, fewer resources are available to process irrelevant auditory input. In this view, more demanding tasks (or a higher task engagement) can protect against distraction (e.g., Sörqvist et al., 2016; Simon et al., 2016; Lavie, 2005). Conversely, *multiple resource theory* (Wickens, 1980) posits that distraction depends not on overall load, but on overlap between processing resources: a sound is more likely to interfere if it draws on the same modality or cognitive process as the ongoing task (Duncan et al., 1997; Samman et al., 2005; Keitel et al., 2013).

Empirical findings support both models under different conditions. For example, Lavie (2005), Sörqvist et al. (2016), and Simon et al. (2016) showed that high cognitive load can reduce distraction in some cases, while others found that cross-modal interference may increase under load (Duncan et al., 1997; Samman et al., 2005; Keitel et al., 2013). These apparent contradictions underscore the need to consider task structure and modality when predicting distractibility.

To conplicate matters further, even under identical task conditions, some people are far more distractible than others. Individual differences research seeks to identify stable traits or abilities that influence how attention is maintained in the presence of competing sounds. A large body of literature emphasizes the critical role of executive control functions. The *Task-Engagement/Distraction Trade-Off* (TEDTOFF) model (Sörqvist and Rönnberg, 2014) highlights working memory capacity (WMC) as a central moderator: individuals with high WMC are more resistant to distraction, likely due to stronger goal maintenance and attentional control (Conway et al., 2001; Kane et al., 2001; Beaman, 2004).

However, not all evidence supports this executive-control-centric view. Macken et al. (2009) present a compelling challenge by arguing that auditory distraction is not primarily due to failures of inhibition or limited WMC. Instead, they show that individuals who are better at globally processing auditory patterns are more susceptible to distraction by task-irrelevant sound. In their study, performance on a global auditory pattern-matching task positively predicted the degree of disruption in a serial recall task when irrelevant sounds were present. In contrast, performance on a deliberate sequence-processing task predicted memory performance but not susceptibility to distraction. These findings suggest that auditory distraction may occur not because of poor attentional control, but because the perceptual system is highly efficient at automatically analyzing sounds, even when they are irrelevant. In this view, distraction results from the strength of perceptual processing rather than a weakness in executive control.

Furthermore, Nagaraj et al. (2020) found that in children, higher WMC did not consistently reduce distraction through noise. Their findings revealed that the magnitude of distraction was not significantly related to WMC. When distraction was quantified as a ratio of errors, high-WMC children sometimes showed even greater vulnerability to noise. These results suggest that irrelevant sounds can gain obligatory access to verbal short-term memory, even in individuals with strong cognitive control, challenging the view that WMC robustly predicts distractibility across conditions.

Personality has also been explored as moderator of distractibility, particularly through its influence on arousal, attentional regulation, and susceptibility to irrelevant input. Findings are, however, mixed. One of the foundational studies in this area by Eysenck and Graydon (1989) found that neurotic introverts, who score high on trait anxiety, were more affected by auditory distraction than stable extraverts. Expanding this perspective, Murphy and Dalton (2014) provide evidence that susceptibility to auditory distraction reflects a stable, trait-like characteristic. In their study, individuals who reported more frequent everyday attentional lapses exhibited significantly greater performance impairments in the presence of a task-irrelevant auditory singleton.

On the other hand, in a large replication effort Bell et al. (2019) found no significant relationship between auditory distraction (measured via changing-state and deviant sound effects) and three broad personality traits: extraversion, neuroticism, and psychoticism.

Finally, a further complexity in assessing individual susceptibility to distraction arises from the fact that subjective reports of distraction do not always align with objective performance measures. While some individuals feel distracted by sounds that produce no measurable performance deficit, others show marked performance impairments without reporting distraction (Paulhus et al., 1990). This dissociation suggests that subjective distraction may be shaped by perceived relevance, personal tolerance, or introspective awareness, rather than by attentional failure *per se*.

In sum, a sound becomes distracting not just because of its physical characteristics, but because of how it interacts with ongoing tasks, individual capacities, and perceptual mechanisms. Understanding distraction requires attention to both interindividual variability and the situational conditions that modulate the cost of attentional capture.

### 2.3 How context shapes auditory relevance

A central limitation of treating sounds as distractors or targets is that it obscures a parallel line of work concerned not with interference, but with *relevance*: why certain sounds are prioritized, interpreted, or remembered even when they do not disrupt ongoing behavior. Across research traditions, this question is addressed under different labels, including stimulus priority, value-driven attention, personal significance, and ecological meaning. What unites these approaches is the assumption that relevance is neither an acoustic property nor a by-product of distraction, but a context-dependent judgment that reflects the listener's goals, learning history, and situational interpretation.

Although distraction and relevance are closely related, they capture different dimensions of auditory attention. Distraction refers to attentional shifts that impose costs on performance or experience, whereas relevance describes the significance attributed to a sound, regardless of whether it helps, hinders, or has no measurable impact on the task at hand. A sound may therefore be highly relevant without being distracting (e.g., a warning signal), or distracting without being relevant (e.g., a sudden but meaningless noise).

While distraction research highlights when and how attention is disrupted, the framework of relevance addresses a different question: how sounds are evaluated in light of goals, expectations, and situational meaning. This perspective brings into focus mechanisms such as auditory object formation, learned significance, and predictive context, which shape not just whether a sound captures attention, but how it is ultimately interpreted. Additionally, it determines whether a sound is appraised as meaningful, useful, or irrelevant.

Contextual relevance is also influenced by how sound is organized percepttually. Object-based models of auditory attention propose that the auditory system organizes incoming information into coherent streams (or auditory objects) based on features such as pitch, timbre, spatial location, and temporal continuity (Shinn-Cunningham, 2008). Attention is not directed to isolated acoustic features but to these higher-order units. Distraction may occur when a competing object captures attention, not because of its raw salience, but because it forms a coherent perceptual unit that competes with the attended stream. Whether an auditory object is further evaluated or ignored as background noise depends on the listener's goals, experience, and task engagement (Parmentier et al., 2008). This highlights the context-sensitive nature of auditory perception.

Context also shapes auditory attention through learned or intrinsic relevance. Certain sounds, such as one's own name or personally meaningful cues, are preferentially processed even when they are task-irrelevant (Roye et al., 2013; Holtze et al., 2021). These stimuli can draw auditory attention not because they are acoustically deviant in a general sense, but because they carry emotional or social meaning. From this perspective, distraction can arise from learned patterns or momentary emphasis of the listener, especially if they occur unpredictably.

Predictive coding accounts further support the idea of auditory attention being sensitive to context. They conceptualize perception as a process of generating and updating internal models of the world (Hughes and Marsh, 2020; Winkler, 2007). In this framework, the brain continuously predicts what it expects to hear. When those predictions are violated, either through unexpected acoustic structure or unexpected meaning, attention is drawn to the resulting prediction error. These prediction errors are shaped by sensory irregularities and higher-level expectations based on task, context, and personal relevance. Auditory attention, in this sense, is not merely reactive but inferential, guided by what the listener anticipates and considers important at a given moment.

Context also plays a role in later stages of attentional processing. In the *three-phase model of involuntary attention and distractibility*, the final stage involves reorienting attention back to the primary task (Escera et al., 2000). Whether this reorientation occurs, and how rapidly it unfolds, depends on how relevant the deviant sound was within the broader context. A sound that is interpreted as highly relevant may delay reengagement with the task, whereas a less meaningful sound may allow for a faster return. This further supports that the consequences of distraction are shaped by the listener's interpretation.

Taken together, these perspectives position relevance as a central organizing principle of auditory attention. Rather than being the opposite of distraction, relevance describes how sounds acquire significance within a given context, shaping prioritization, interpretation, and attentional persistence even in the absence of overt performance costs.

### 2.4 The dynamics of attention

As outlined in the preceding sections, auditory attention is not a static state. It fluctuates over time, shaped by neural rhythms, mental fatigue, and adaptive mechanisms that modulate how sounds are processed. These fluctuations can profoundly influence how a sound is experienced. This section highlights how attentional engagement varies moment to moment, and how such variability contributes to the evaluation of sound.

Recent studies suggest that attentional engagement waxes and wanes across multiple timescales. At ultra-slow frequencies (0.07 Hz), Kasten et al. (2024) identified rhythmic shifts between externally and internally oriented processing during sustained auditory attention. These shifts, reflected in fluctuations in speech tracking and alpha-band activity, predicted task performance: listeners were more likely to detect auditory targets during externally focused states. Similar oscillatory dynamics have been observed in the theta band. Lui et al. (2023) demonstrated that the phase of ongoing theta oscillations modulates perceptual sensitivity to distractors in a pitch discrimination task. Together, these findings suggest that the evaluation of sound is not constant but varies with rhythmic fluctuations in neural excitability, even when the task and input remain constant.

Adaptation and habituation further modulate how sounds are processed over time. Altmann et al. (2008) showed that listeners quickly learn to predict patterns in natural sound sequences, with Mismatch Negativity (MMN) amplitudes decreasing as exposure to statistical regularities increases. Similarly, Herrmann et al. (2013) found that the tuning of frequency-specific responses in auditory cortex changes with recent spectral context, broadening or narrowing depending on the variability of the input. In addition to short-term adaptation, there is also evidence for longer-term habituation processes that reduce the neural responsiveness to repeated auditory input over extended exposure. Mortezapouraghdam et al. (2016) applied a Bayesian model to track trial-wise changes in the phase concentration of the N1 component across hundreds of tone presentations. They found that soft, low-salience sounds evoked a gradual loss of phase synchronization over time, interpreted as attentional unbinding due to habituation. In contrast, louder, aversive sounds maintained stable phase alignment, suggesting continued attentional monitoring.

In addition to rhythmic fluctuations, mental fatigue reflects a gradual, non-cyclical form of temporal variability in auditory attention. In a prolonged listening task, Moore et al. (2017) observed reduced N1 amplitudes and decreased parietal activity over time, alongside higher self-reported fatigue. These changes were interpreted as signs of declining sensory gain and effortful disengagement, indicating that sustained auditory processing may become less efficient as attentional resources are depleted.

The dynamic allocation and recovery of attention can also be traced in event-related potentials. Mandal et al. (2024) demonstrated that attention can be temporarily misallocated to a salient, task-irrelevant auditory distractor before being reoriented toward a target, as indexed by the N2ac component. Using an auditory search task, they showed that only more salient distractors elicited early N2ac responses and delayed attention to the target, consistent with true attentional capture. Less salient distractors caused behavioral delays without corresponding neural markers of attentional capture, suggesting filtering costs instead. This dynamic redirection suggests that auditory attention is not a fixed focus but an actively regulated process. Taken together, these findings underscore the temporal variability of auditory attention. Neural fluctuations, fatigue, and adaptation shape how attentional resources are deployed. Even when acoustic and contextual features, and intentional factors remain stable, the listener's attentional state can modulate how a sound is processed.

### 2.5 Interim summary

Together, the preceding sections outline a multidimensional view of auditory experience, where the evaluation of sound arises through the interaction of acoustic input, contextual factors, and the listener's characteristics. Across theoretical perspectives, including novelty-based alerting responses, predictive inference, and goal conflict, distraction is described as an adaptive process that reflects the brain's responsiveness to meaningful change.

This helps address the central question of this review: How do listeners judge whether a sound matters in everyday life? The reviewed models suggest that such judgments are not determined by the sound alone, but emerge from how it is interpreted in relation to goals, expectations, and situational demands. In this view, relevance and distraction are outcomes of a shared attentional process shaped by context. Table 1 summarizes several key models that have shaped our understanding of auditory attention. Each model highlights a different mechanism and entry point into the attentional system, but they all converge on a common theme: the significance and cost of a sound cannot be fully explained by its physical characteristics alone.

**Table 1:** Theoretical models of auditory distraction and relevance.

**Model**	**Mechanism emphasized**	**Trigger**	**Interpretation focus**
Orienting reflex	Novelty detection and alerting response	Unexpected stimuli that violate an internal model	Neutral evaluation of change; system readiness to respond
Three-phase model	Sequential ERP stages (MMN, P3a, RON)	Auditory deviance in a stream of regular input	Structured neural response indicating temporary reallocation of attention
Duplex mechanism	Attention capture vs. task interference	Salient or deviant input, especially in demanding tasks	Behavioral cost or neutrality depending on goal alignment
Predictive coding	Prediction error signaling within a generative model	Mismatch between expected and actual input	Inference-based evaluation of sensory significance

## 3 Studying auditory distraction and relevance: methodological considerations

Gaining insight into how sounds are experienced requires more than simply identifying whether an attentional shift has occurred. It calls for methodological approaches capable of capturing the complex and context-sensitive dynamics of auditory attention, as well as the subtle ways in which listeners engage with sound in natural settings. As outlined in the preceding sections, distraction and relevance are not inherent features of acoustic stimuli, but rather outcomes that emerge through the interaction of sensory input, cognitive goals, internal states, situational context and temporal dynamics, often without an explicit task structure that would fix what counts as “relevant” or “distracting.” This raises central questions: under what conditions does a sound become disruptive, when is it evaluated as meaningful, and how can subjective reports, behavior, brain measures, and acoustic descriptions converge or diverge in addressing these outcomes? No single approach can on its own capture the unfolding and multifaceted processes through which a sound acquires meaning or disruptiveness. An integrated account of auditory attention therefore requires not only temporal and spatial precision, or ecological validity, but also a critical understanding of what different data types can and cannot reveal about the processes under investigation.

A useful way to organize these approaches is to distinguish four complementary levels at which the same listening episode can be described. Acoustic analyses characterize the proximal soundscape, i.e., the physical structure of the auditory input at the listener. Brain-based measures index the neural representation of that soundscape, capturing how auditory information is encoded and dynamically selected over time. Subjective reports provide access to the perceptual soundscape, understood here as the listener's appraisal and experienced meaning of sounds in context. Behavioral measures reflect the measurable consequences of listening, such as performance costs, compensatory effort, or task-level benefits. These levels are related but not interchangeable; dissociations are expected and often informative, particularly in naturalistic settings where goals and contextual reference frames are implicit and variable.

In the following subsections, we examine several widely used approaches to studying auditory attention in general and the evaluation of sound in particular, including acoustic modeling, subjective reports, behavioral performance and brain-based measures. Each of these methods offers distinct insights into the mechanisms of distraction and relevance, but also presents important limitations. A comprehensive account of auditory attention requires not only temporal and spatial precision, or ecological validity, but also a critical understanding of what different types of data can and cannot reveal about the processes under investigation.

### 3.1 Acoustic modeling: describing the soundscape

Because the acoustic input constrains what can be perceived and selected, we first consider methods that characterize the physical properties of sound. Acoustic modeling quantifies what the soundscape offers at the level of physical description: its intensity, structure, and potential salience. In doing so, it focuses on the signal rather than on the listener. These features form a crucial foundation for understanding auditory distraction and relevance, but their interpretation depends entirely on the listener's state and context. On their own, they identify potential triggers, not outcomes.

Measures such as sound pressure level (SPL), modulation depth, signal-to-noise ratio (SNR), and indices of spectral or temporal salience quantify how sounds differ from their background, often identifying events that may be perceptually prominent (e.g., Hong et al., 2020; Thoret et al., 2020). More advanced frameworks, including computational auditory scene analysis and spectral novelty detection, model how the auditory system might parse and respond to complex acoustic environments (Gutschalk and Dykstra, 2014; Müller, 2021). These tools enable researchers to detect signal changes, structure, and regularity across extended, naturalistic recordings without requiring human input during data acquisition.

One of the central strengths of acoustic models lies in their scalability and their relatively standardized, reproducible quantification of sound structure. They are data-driven and non-invasive, making them especially suited to the analysis of large, continuous soundscapes such as urban noise or open-plan office environments. They are also reproducible across studies and settings, offering standardized ways to quantify acoustic dynamics. These metrics frequently serve as regressors in neural models, for instance in EEG studies, where they are used to examine how the brain tracks or responds to specific acoustic features over time (e.g., Haupt et al., 2025; Mirkovic et al., 2015). In this way, acoustic modeling constitutes a valuable methodological bridge between environmental input and neural response.

At the same time, acoustic modeling remains limited in what it can reveal about perceptual or cognitive processes. While these models describe the acoustic dynamics of an environment, they do not capture how listeners interpret or respond to sound under varying task demands or mental states (Kothinti and Elhilali, 2023). This limitation becomes especially consequential in naturalistic settings, where the task reference frame that defines what counts as “relevant” or “distracting” is often implicit or shifting. A computationally salient event may be perceptually ignored due to habituation, selective attention, or contextual irrelevance, while an acoustically unremarkable sound may elicit attentional capture if it is personally meaningful. These models also rely on implicit assumptions about which features are likely to attract attention—assumptions that may not generalize across individuals or settings. Crucially, acoustic modeling provides no information about subjective experience, task engagement, or listener goals. Without behavioral or neural validation, there is a risk of overinterpreting signal-based measures as proxies for relevance or distraction. To understand whether a sound is processed as meaningful or disruptive, acoustic models must be integrated with methods that index attention more directly.

### 3.2 Subjective reports: accessing listener experience

While acoustic modeling characterizes the acoustic structure of an environment, it cannot on its own determine how that structure is perceived, interpreted, or evaluated by a listener. To capture this subjective dimension, we turn to methods that ask participants directly about their perceptions and evaluations. Subjective reports provide access to the emotional and contextual dimensions of experiencing sound. They are uniquely positioned to capture the listener's appraisal of a sound—its perceived valence, motivational significance, and situational relevance—all of which may be overlooked by more externally focused measures.

Questionnaires, rating scales, verbal feedback, and ecological momentary assessment (EMA), which collects self-reported data in real time in natural environments (Shiffman et al., 2008), including techniques such as experience sampling, allow researchers to collect self reported data on whether, when, and how a sound was noticed, interfered with a task, or was considered distracting. These methods are particularly well suited to studies conducted outside of traditional laboratory environments, where understanding how listeners perceive and interpret sound within real life contexts is central (e.g., Yoon et al., 2014; Kjellberg et al., 1996). This is especially important outside the laboratory, where the task reference frame that defines what counts as “relevant” or “distracting” is often implicit, variable, or divided across competing goals.

One of the principal strengths of subjective measures is their ability to capture the internal perspective of the listener, which is not necessarily represented by standardized measures (Kuchinsky et al., 2020; Paulhus et al., 1990). Unlike behavioral or neural data, which must be interpreted through external proxies, subjective reports allow researchers to directly assess how a sound was experienced from the listener's perspective (Farkas et al., 2016). These insights are especially valuable in complex auditory environments, where attention is influenced by mood, motivation, fatigue, and prior knowledge (Pang et al., 2019; Asutay and Västfjäll, 2012; Gustafson et al., 2018). In such contexts, subjective accounts can help explain why similar acoustic events might be ignored in one instance and noticed or evaluated differently in another.

Subjective methods are highly adaptable: they can be administered during or after a task, used in controlled laboratory experiments or field studies, and tailored to assess constructs ranging from perceived effort and annoyance to emotional resonance and situational awareness (Verhagen et al., 2016; Seifert et al., 2018). In mobile and ecologically grounded research, digital tools such as smartphone-based experience sampling make it feasible to collect subjective data in context with minimal disruption to ongoing activity (e.g., Randall and Rickard, 2013; Seifert et al., 2018; Holube et al., 2020). EMA protocols can also be refined to improve ecological validity and participant compliance. For example, Mansour et al. (2021) introduced a guided EMA design in which participants completed structured listening tasks in both real-world and virtual environments, and found that subjective ratings remained consistent across conditions even in acoustically complex contexts.

However, subjective reports also carry important limitations. They rely on introspection and memory, both of which are influenced by biases in recall, interpretation, and social desirability (Houtveen and Oei, 2007; Latkin et al., 2017). Listeners may report only those experiences that were most intense or emotionally charged, or they may reinterpret past experiences in light of subsequent events (Stone et al., 1998; Houtveen and Oei, 2007; Raphael and Cloitre, 1994). This is consistent with the peak-end rule, which shows that retrospective evaluations are disproportionately influenced by the most intense (peak) and final (end) moments of an experience, while the overall duration or average intensity is often neglected (Kahneman et al., 1993; Alaybek et al., 2022). A sound that momentarily shifted attention may not be remembered at all, while a sound that was only faintly disruptive may be overemphasized in retrospect. Moreover, the act of asking about sound can itself draw attention to it, altering the listener's baseline perception (c.f. Hjortskov, 2017).

Perhaps most importantly, subjective reports do not always align with behavioral or neural indicators of attention (e.g., Macdonald et al., 2011). Such dissociations do not imply that subjective reports are wrong; they indicate that experiential appraisal and performance costs can vary independently. Listeners may report feeling distracted without any observable decline in performance, or they may perform poorly without attributing their errors to external sound. In this sense, subjective and objective measures capture different aspects of attentional processing. Self-report reflects conscious experience and evaluation of listening (e.g., noticing, annoyance, perceived effort), whereas behavioral and neural measures reflect its observable consequences. Neither can fully substitute for the other, but together they help to illuminate how listeners interpret, adapt to, and evaluate sound in context.

### 3.3 Behavioral measures: capturing performance outcomes

While subjective reports reflect how a sound is experienced, behavioral measures reveal how it affects task performance. They offer insight into the consequences of attentional shifts—particularly when a sound disrupts, supports, or goes unnoticed during goal-directed activity. Behavioral measures are therefore most informative about distraction when attentional shifts translate into measurable performance costs.

Measures such as response time, accuracy, error rates, and performance variability are commonly used to infer how listeners respond to competing auditory input (e.g., Parmentier et al., 2010; Li et al., 2013; Schröger and Wolff, 1998; Max et al., 2015). In experiments, such changes are often interpreted as evidence of attentional reallocation. For example, slower responses or increased errors may reflect distraction from an additional sound, while stable or improved performance may suggest that a sound was effectively integrated, ignored, or even supportive of task engagement. Numerous studies using oddball paradigms have shown that rare or deviant sounds, especially those conveying task-relevant cues, can slow reaction times or increase error rates, particularly when they violate the listener's learned expectations (Parmentier et al., 2010; Li et al., 2013). However, if the deviant sound provides useful information (e.g., about task timing), it may facilitate performance instead of hindering it (Parmentier et al., 2010).

One of the strengths of behavioral measures lies in their focus on observable outcomes and versatility (Henriques and Michalski, 2019). They are relatively simple to collect, applicable across a wide range of tasks and environments, and easy to compare across individuals and conditions (e.g., Satish and Streufert, 1997; Henriques and Michalski, 2019). Behavioral performance can indicate how listeners prioritize, suppress, or adapt to sound in real time. In studies of auditory attention, these measures are often used to evaluate how changes in the acoustic environment, task demands, or listener state influence ongoing performance. For instance, embedding distracting auditory changes within the same perceptual object as task-relevant stimuli can amplify distraction effects, revealing how deeply intertwined perceptual and attentional processes are (Schröger and Wolff, 1998). Likewise, predictable changes in the sound stream are less disruptive when their structure is known or task-irrelevant, showing that cognitive expectations modulate the behavioral cost of distraction (Max et al., 2015).

At the same time, behavioral data offer only indirect access to the internal processes that shape attentional outcomes. They are most informative at the level of task consequences rather than subjective evaluation. A change in performance may indicate that a sound was processed differently, but it cannot distinguish how that sound was evaluated by the listener (Kaiser et al., 2018). Likewise, a lack of behavioral change does not necessarily imply that attention remained unaffected. Listeners may compensate for momentary attentional shifts, or they may register a sound without it influencing performance in measurable ways. Behavioral responses are also shaped by factors such as strategy, fatigue, motivation, and individual variability in cognitive control, making it difficult to isolate attention from other influences. For example, older adults and children often exhibit increased behavioral distraction, but these effects may reflect broader age-related differences in reorienting efficiency or attentional restoration rather than auditory sensitivity *per se* (Horváth et al., 2009).

Moreover, stable performance can mask compensatory control or increased effort, as listeners invest additional resources to maintain accuracy despite interference (Graydon and and Eysenck, 1989). Over time, this can result in mental fatigue or strain, which behavioral measures alone may miss. Furthermore, attention can be captured without conscious awareness, especially under high cognitive load. Individuals may be unable to reflect on or report the distraction, even as it influences processing (Craik, 2014; Anderson, 2024). For these reasons, behavioral outcomes provide only a partial picture of attentional effects. Therefore, they are most informative when combined with complementary methods, such as neural measures (e.g., ERPs) that can index attentional capture independently of overt behavior (Jankowiak and Berti, 2007).

### 3.4 Brain-based measures: indexing auditory attention in the brain

Some attentional shifts occur rapidly and may not reach conscious awareness. Brain-based measures are critical for detecting these early and covert processes. By capturing the timing and strength of neural responses, they provide access to attentional engagement and arousal-related dynamics that are not always visible in behavior or self-report. These methods reveal not only the degree of neural activation linked to alertness, but also how attentional focus on sound is modulated depending on situational relevance or cognitive state. Neural data thus help illuminate when attention shifts occur and how strongly auditory input is processed under different contextual and congitive states.

Different methods contribute complementary strengths. EEG and magnetoencephalography (MEG) offer millisecond-level temporal resolution, allowing researchers to observe when attention is captured or redirected. Functional magnetic resonance imaging (fMRI), by contrast, provides high spatial precision and helps localize the brain regions involved in auditory attention. Studies combining these techniques have shown that shifts in auditory attention can occur within a few hundred milliseconds after a stimulus, and that distinct brain areas, such as the supratemporal plane, parietal cortex, and inferior frontal gyrus, are engaged depending on the type and context of the sound (Brunetti et al., 2008; Polomac et al., 2015).

Crucially, brain-based measures can detect attentional responses that are not always reflected in behavior or subjective reports. For instance, increased gamma-band activity in auditory and frontal brain regions has been observed during demanding listening tasks, even when performance remains stable (Polomac et al., 2015). Neural data can also reveal how attention is distributed across competing auditory sources, such as in studies of selective listening, where patterns of synchrony between auditory cortex and parietal regions reflect the listener's focus (Huang et al., 2014; Walz et al., 2013). Because neural signals reflect ongoing processing rather than *post hoc* interpretation, they offer a unique window into both early and sustained stages of attention, even in the absence of an overt response.

Although neural activity cannot on its own indicate whether a sound is experienced as distracting or relevant, it can nonetheless help contextualize behavioral or subjective outcomes. Studies combining brain-based measures with task performance or self-report—both in laboratory and semi-naturalistic settings—show that fluctuations in neural markers of attentional engagement, such as alpha or theta dynamics, may precede lapses in sustained performance or increases in perceived effort (e.g., Zink et al., 2016; Borghini et al., 2014). Likewise, variations in cortical tracking of attended speech have been linked to differences in comprehension during more naturalistic listening (Mirkovic et al., 2015). In this way, brain-based measures do not determine how a sound was evaluated, but they can illuminate how moment-to-moment changes in attentional state contribute to observable or felt outcomes.

Recent developments in mobile and wearable EEG have further extended the applicability of brain-based methods (e.g., Debener et al., 2012; Bleichner et al., 2016; Crétot-Richert et al., 2023). Wireless EEG and ear-centered systems allow researchers to study neural correlates of auditory attention in ecologically valid settings over extended time periods (Hölle and Bleichner, 2023). These approaches open the door to understanding how attention operates in the dynamic flow of everyday life, while still retaining access to time-resolved neural data. However, they also introduce new challenges related to data quality, artifact management, and the integration of concurrent data streams such as audio, behavior, and movement (Kappel et al., 2017).

Despite these advantages, brain-based measures carry an important interpretive limitation: they cannot by themselves reveal how a sound was subjectively evaluated. A neural response may indicate that a sound was processed or attended to, but not how it was experienced. For instance, increased activity in auditory cortices or synchronization between auditory and frontal regions can occur in both distracting and goal-facilitating contexts (e.g., Polomac et al., 2015; Brunetti et al., 2008). In this way, brain-based methods index the occurrence and timing of attentional shifts, but not its valence or perceived meaning. Although some studies have demonstrated that emotional valence can be decoded from EEG data using machine learning and advanced signal processing approaches (e.g., Apicella et al., 2021), these methods typically rely on highly controlled paradigms, substantial preprocessing, and averaging across trials, which limits their applicability in ecologically valid auditory contexts. This ambiguity is especially problematic in ecologically valid settings, where the perceived meaning of sound is deeply context-dependent. Without complementary behavioral or subjective data, it is difficult to determine how an observed change in neural activity relates to the subsequent evaluation of sound. Moreover, unlike questionnaires or behavioral tasks that can capture responses to single events, neural measures such as EEG typically require averaging across many trials to extract reliable signals, which limits their ability to index responses to individual sounds.

Nonetheless, brain-based approaches remain essential for a comprehensive account of auditory attention. They allow researchers to trace the timing and coordination of neural activity that underlies listening, even in the absence of overt signs. When used in conjunction with behavioral and subjective methods, they help illuminate not just when attention shifts occur, but also how listeners adapt to and interpret sound in everyday settings (Salmela et al., 2018; Walz et al., 2013).

### 3.5 Practical considerations for real-world data collection

Field-based studies introduce several practical and ethical challenges that differ from traditional laboratory paradigms. First, continuous audio or environmental recordings may inadvertently capture identifiable speech or personal information, requiring strict adherence to data-minimization principles, transparent consent procedures, and secure storage practices. Second, mobile and wearable systems often rely on multiple devices (e.g., EEG, microphones, accelerometers), making reliable synchronization essential; issues such as clock drift, timestamp loss, or environmental variability can complicate alignment across data streams. Third, sharing naturalistic datasets is inherently constrained by privacy considerations, as raw audio or video often cannot be made publicly available. In many cases, ethically responsible data sharing requires releasing only de-identified features, annotations, or derived measures rather than the full recordings. Acknowledging these constraints is crucial for developing robust and reproducible research practices as the field moves toward increasingly ecological methodologies. To complement these considerations, Table 2 summarizes a selection of existing tools and standards that support responsible and reproducible real-world data collection. These examples are not exhaustive but illustrate commonly used frameworks that facilitate privacy-aware recording, multimodal synchronization, and structured data organization.

**Table 2:** Best-practice considerations for real-world auditory data collection.

**Challenge area**	**Best-practice examples**	**Purpose/benefit**
Privacy and data governance	Data minimization; on-device processing; selective redaction tools, e.g., Audio Feature Extraction (AFEx) (Hölle et al., 2022)	Reduce collection and storage of identifiable speech/audio; improve compliance with ethical and legal requirements
Synchronization across devices	Hardware timestamping; Lab Streaming Layer (LSL) (Kothe et al., 2025)	Align EEG, audio, motion, and behavioral data streams; reduce clock drift and integration errors
Standardized data organization	Structured metadata standards such as BIDS (Brain Imaging Data Structure) (Gorgolewski et al., 2016)	Ensure reproducibility; facilitate long-term storage and sharing of de-identified derivatives

### 3.6 Interim summary

As shown across the preceding sections, each methodological approach captures different facets of the evaluation of sound: subjective reports access lived experience, behavioral measures reflect performance outcomes, brain-based methods trace neural engagement, and acoustic modeling characterizes the stimulus environment. Individually, each offers valuable insights—but in combination they provide a more complete account of the multifaceted and context-sensitive nature of auditory attention. This is essential for addressing the central question of this review: How do listeners judge whether a sound matters in everyday life, where the reference frame that defines what counts as “relevant” or “distracting” may be implicit and variable? Because these evaluations depend on affective tone, cognitive goals, and situational framing, understanding auditory relevance beyond the laboratory benefits from methodological integration that reflects this complexity. Table 3 gives a brief summary of the methods discussed, with their strengths and limitations.

**Table 3:** Comparative strengths and limitations of methods used to study auditory attention.

**Method**	**Captures**	**Strengths**	**Limitations**
Subjective reports	Perceived relevance and personal experience	Access to internal state; ecologically valid; suitable for real-world studies	Subject to memory bias, social desirability, and context effects; may not align with behavior or brain data
Behavioral measures	Task performance (e.g., response time, accuracy)	Objective and quantifiable; real-time effects; easy to deploy across tasks	Does not reveal how sounds were experienced; compensatory strategies may mask effects
Brain-based measures	Neural responses to sound and attention shifts	High temporal resolution (EEG/MEG); detects covert processing; insights into timing and mechanism	Ambiguous interpretation without behavioral/contextual data; ecological validity may be limited
Acoustic modeling	Stimulus salience and structural properties	Scalable; reproducible; useful for characterizing complex environments	No information about listener state or experience; relevance must be inferred

## 4 Integrating perspectives: toward a comprehensive understanding of auditory relevance and distraction

### 4.1 Distraction and relevance as outcomes of the same process

In practice, attention is frequently described as either focused successfully on a task, implying relevance, or disrupted by an external event, implying distraction. But how do these judgments arise in everyday listening? When does a redirection of attention support ongoing goals, and when is it experienced as interference? Rather than treating relevance and distraction as distinct categories, we argue that they reflect different outcomes of attentional reorienting and selection processes.

From this perspective, both relevance and distraction emerge from the brain's ongoing evaluation of incoming sensory input in relation to current goals and internal states. The difference lies not in the attentional process itself, but in how a shift in attention is interpreted. A redirection of attention may be judged as helpful, appropriate, or meaningful, in which case it is seen as relevant, or it may be perceived as disruptive or unnecessary, and thus considered a distraction.

This framing highlights that distraction is not necessarily a failure of attention but a function of it. The capacity to shift attention in response to changing environmental demands is one of the core features of an adaptive attentional system. As Hughes (2014) describes, consider the example of working in an office while a colleague is speaking just outside your door. You may manage to ignore the conversation and remain focused. But if the same colleague suddenly shouts “Fire!”, your attention is immediately redirected. The attentional mechanism is the same in both situations. What changes is the context and whether the shift is judged as relevant or distracting (Hughes, 2014).

This review therefore suggests that the commonly made distinction between “distracting” and “relevant” sounds, while methodologically useful, in laboratory paradigms that define these categories via explicit task goals and outcome measures, may oversimplify the more flexible and interpretive nature of auditory attention in daily life. Listeners encounter sounds that defy neat categorization: some draw attention without affecting performance, others are technically relevant but emotionally aversive, and many fluctuate in their impact depending on context and internal state. Rather than assigning fixed roles to sounds based on task demands, it may be more productive to examine how listeners construct judgments about them. To capture this interpretive flexibility, we propose a multidimensional framework.

### 4.2 A multidimensional perspective on auditory experience

We suggest that auditory experiences can be understood along three interacting dimensions: arousal, valence, and contextual framing. Arousal captures the activating potential of a sound, whether it alerts, excites, or soothes. Valence refers to its emotional tone, ranging from pleasant to unpleasant. Contextual framing encompasses the situational and personal backdrop against which a sound is interpreted, including task relevance, social meaning, fatigue, motivation, or mood. We intend these dimensions as a heuristic for organizing everyday evaluations of sound, not as a process model that specifies processing stages or causal directionality of attentional selection.

This multidimensional framework is motivated by limitations in traditional task-centric and stimulus-driven models. Predictive coding and ERP-based approaches offer valuable mechanistic insights, but they tend to overlook the affective and situational richness of real-world listening. These models typically emphasize statistical or physiological features of the stimulus, such as how surprising a sound is relative to prior expectations, or how quickly it evokes a neural response. While these accounts have advanced our understanding of deviance detection and sensory encoding, they often emphasize stimulus-driven regularities and can underrepresent how goals and situational meaning shape the evaluation of sound in everyday contexts.

In contrast, contextual framing, drawing from appraisal theory (cf. Moors, 2014), highlights that the meaning of a sound is actively constructed by the listener. This construction depends on a range of internal and external factors, including current goals, mood, prior experience, and social setting. As a result, the same sound may elicit very different reactions depending on how it is interpreted in context. Rather than displacing mechanistic models, this listener-centered perspective complements them by adding a flexible interpretive layer that better reflects the complexity of everyday auditory experience.

By drawing on dimensional models of affect (e.g., Schimmack and Grob, 2000; Korsmit et al., 2023) and appraisal-based accounts of emotional salience (Pessoa, 2009; Phan et al., 2004), this framework also connects auditory cognition with broader theories of how organisms prioritize information in emotionally and contextually meaningful ways. It supports a shift from viewing attention as solely stimulus-driven to understanding it as dynamically shaped by affective state and situational relevance.

At the same time, these three dimensions can covary and interact (cf. Pessoa, 2009). Arousal and valence often interact in non-linear ways; highly arousing sounds can be either pleasant or unpleasant, and their subjective intensity may vary depending on contextual expectations. Contextual framing itself is not a static backdrop but evolves with the listener's emotional and physiological state.

Individual listener characteristics and environmental settings can systematically shape how the three proposed dimensions unfold. For example, age-related changes in hearing acuity, cognitive control, or auditory scene analysis can increase the arousal or aversiveness of sounds that younger adults experience as neutral (Peelle and Wingfield, 2016; Hake et al., 2025). Even mild hearing loss can alter neural and emotional responses to sound, leading to reduced valence sensitivity and increased listening effort (Picou et al., 2021). Fatigue and negative mood similarly bias valence judgments, making listeners more susceptible to perceiving neutral or mildly deviant sounds as distracting or aversive (Asutay and Västfjäll, 2012).

Similarly, contextual framing strongly determines emotional evaluation: the same acoustic features may evoke different affective responses depending on the setting. For instance, speech or music judged neutral or pleasant in one environment may be experienced as aversive or distracting in another, depending on contextual relevance and expectations (Liuni et al., 2020; Kim and Howie, 2021). For example, in an open-plan office, speech may be evaluated as distracting, whereas in a clinical or social environment, the same acoustic cues may be interpreted as meaningful or socially relevant.

These examples illustrate that arousal, valence, and contextual appraisal are not fixed acoustic properties but instead emerge dynamically from the interaction between acoustic input, listener-specific factors (age, cognition, mood, hearing status), and situational demands. Therefore, our framework should be understood as a heuristic that captures the layered, recursive, and interpretive nature of auditory experiences.

This perspective also implies that in naturalistic settings researchers must either re-specify the contextual reference frame that gives “relevance” and “distraction” their meaning, or treat these labels as provisional and contingent on goals, constraints, and momentary demands.

Together, these dimensions offer a more flexible, listener-centered vocabulary for describing real-world listening. They also inform experimental design and methodological triangulation, for instance by encouraging the integration of subjective reports, physiological markers, and acoustic modeling. To illustrate how this framework can be operationalized across different empirical approaches, Table 4 provides examples of measurable indicators associated with each dimension. These indicators are intended as illustrative examples rather than an exhaustive or prescriptive list. Numerous physiological, behavioral, and neural measures can index arousal, valence, or contextual framing, and the most appropriate choice always depends on the specific research question, methodological constraints, and the ecological context of the study.

**Table 4:** Dimensions of auditory experience and example measurable indicators.

**Dimension**	**Description**	**Measurable indicators (examples)**
Arousal	Physiological activation or alerting response to sound	Pupil dilation; alpha suppression; heart rate deceleration
Valence	Emotional or affective evaluation of the sound	Self-reported pleasantness/aversiveness; late positive potential (LPP); amygdala activation
Contextual framing	Task goals, expectations, personal meaning, environmental context	Location/movement data; experience sampling reports; EEG indicators of expectation violations (e.g., MMN)

**Figure 1:**
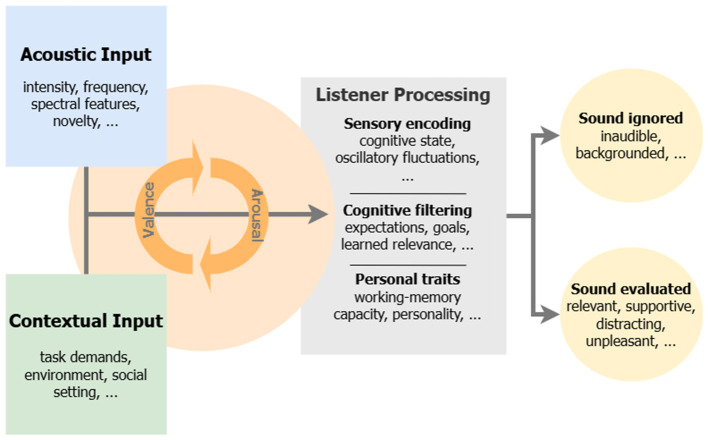
A multidimensional model of auditory experience in the real world. Acoustic features and contextual factors interact with affective dimensions (arousal and valence) and listener characteristics to shape the interpretation of sound. Everyday sounds can occupy different positions along these dimensions depending on situational framing. For example, a phone notification may be experienced as helpful in one moment (e.g., an expected message) but distracting in another (e.g., during focused work), and even a nominally high-arousal sound such as an alarm can be perceived as reassuring or aversive depending on context. These examples illustrate that the interpretation of a sound is not fixed but emerges from dynamic interactions across acoustic, affective, cognitive, and contextual domains.

Figure 1 visualizes this perspective, illustrating how acoustic and contextual characteristics feed into listener processing, dynamically modulated by arousal and valence. How a listener evaluates a sound emerges from this ongoing integration.

### 4.3 Implications for future research

This article argues that concepts such as distraction and relevance are not neutral descriptors of sound, but scientific constructs that have been operationalized primarily within laboratory reference frames—typically through explicit task goals, stimulus roles (target vs. non-target), and performance-based outcomes. Within such paradigms, these constructs enable precise inference: laboratory studies can isolate mechanisms, quantify effects, and support strong causal conclusions about when and how a sound disrupts performance or facilitates goal-directed processing. However, outside these tightly specified frames, the same labels become increasingly context-dependent and therefore harder to apply without additional information about goals, expectations, and situational constraints. In everyday listening, a sound may simultaneously support one goal and disrupt another, and the distinction between “distractor” and “relevant signal” can shift over time within the same environment. Future research should therefore either (i) explicitly re-specify the reference frame that makes these constructs interpretable in natural settings, or (ii) acknowledge where laboratory-derived labels lose explanatory power when detached from their original operational definitions.

Progress lies in systematically bridging the gap between laboratory operationalizations of distraction/relevance and the more weakly specified reference frames of real-world listening. We advocate for incremental steps toward ecological validity. Rather than moving directly from simplified lab tasks to unconstrained field contexts, researchers should gradually introduce complexity, for example, by relaxing constraints on task demands, environmental variability, or participant behavior. This stepwise approach enables researchers to identify where laboratory-derived findings hold, where they diverge, and why, providing a more nuanced understanding of auditory attention across contexts.

Careful formulation of research questions is essential when designing these intermediate steps. In natural settings, this risk is amplified because the reference frame that defines “relevance” or “distraction” (goals, expectations, competing demands) is often implicit and may vary across moments. Each method captures different aspects of auditory processing, and contradictory findings often reflect differences in methodological scope rather than true inconsistencies. By aligning the research question, context, and measurement tools—whether behavioral, neural, subjective, or acoustic—researchers can ensure that results remain interpretable and comparable across levels of ecological validity.

To illustrate how methodological pluralism can be implemented in practice, Table 5 presents three example study designs that combine different methods across levels of ecological realism. These examples demonstrate how complementary tools can be aligned to capture early neural responses, task-level performance, and real-world contextual evaluation within a unified research program.

**Table 5:** Example study designs illustrating methodological pluralism across levels of ecological validity.

**Design type**	**Example setup**	**Methods combined**	**Insights provided**
Controlled laboratory task	Oddball paradigm with controlled auditory deviants	EEG, reaction times, accuracy	Early neural markers of attentional capture under tightly constrained conditions; strong causal inference
Semi-naturalistic dual task	Speech-in-noise listening while performing a concurrent visuomotor task	EEG, behavioral performance, subjective effort ratings	Sharing of attention across modalities; interactions between task load, perceptual effort, and distraction in more realistic environments
Real-world recording	Mobile EEG with ecological momentary assessment during daily activities	Mobile EEG, EMA surveys, passive audio recording	Moment-to-moment attentional fluctuations; contextual framing; subjective evaluations of sound in natural settings

Emerging technologies, such as mobile neurophysiology, digital experience sampling, and wearable sensors, now enable studying auditory attention across naturalistic settings and extended timescales (e.g., Hölle and Bleichner, 2023; Paas et al., 2024; Verhagen et al., 2016). These advances, combined with methodological triangulation, allow researchers to link behavioral, neural, subjective, and acoustic data within the same individuals and situations. A central message of this review is that these approaches often address the same underlying process from different levels of description: acoustic analyses quantify structure in the environment, neural measures index processing and engagement, behavior reflects task-level consequences, and subjective reports capture evaluation and meaning.

Importantly, divergences between methods should not be treated as failures but as informative: they can reveal which aspects of the process are expressed at one level of description but not another, or under which contexts different operationalizations converge. Methodological pluralism is therefore not a call to replace established approaches, but a guide for interpreting what each method can—and cannot—tell us about relevance and distraction in everyday listening.

Single-method studies remain indispensable, providing the foundational insights on which integrative approaches build. However, the complexity of auditory attention in real life calls for designs that make the mapping between levels explicit. By systematically examining when findings converge across measures and when they diverge, future research can better capture the felt and situated experience of listening and explain how sound becomes meaningful—and why it sometimes disrupts, supports, or transforms our engagement with the world.
